# Purkinje cell BKchannel ablation induces abnormal rhythm in deep cerebellar nuclei and prevents LTD

**DOI:** 10.1038/s41598-018-22654-6

**Published:** 2018-03-09

**Authors:** Guy Cheron, Javier Márquez-Ruiz, Julian Cheron, Cynthia Prigogine, Claudia Ammann, Robert Lukowski, Peter Ruth, Bernard Dan

**Affiliations:** 1 Laboratory of Electrophysiology, Université de Mons, Mons, Belgium; 2 Laboratory of Neurophysiology and Movement Biomechanics, ULB Neuroscience Institute, Université Libre de Bruxelles, Brussels, Belgium; 3 División de Neurociencias, Universidad Pablo de Olavide, Sevilla, Spain; 4 Department of Pharmacology and Toxicology, Institute of Toxicology & Clinical Pharmacy, Universität Tübingen, Tübingen, Germany; 5 Inkendaal Rehabilitation Hospital, Vlezenbeek, Belgium

## Abstract

Purkinje cells (PC) control deep cerebellar nuclei (DCN), which in turn inhibit inferior olive nucleus, closing a positive feedback loop via climbing fibers. PC highly express potassium BK channels but their contribution to the olivo-cerebellar loop is not clear. Using multiple-unit recordings in alert mice we found in that selective deletion of BK channels in PC induces a decrease in their simple spike firing with a beta-range bursting pattern and fast intraburst frequency (~200 Hz). To determine the impact of this abnormal rhythm on the olivo-cerebellar loop we analyzed simultaneous rhythmicity in different cerebellar structures. We found that this abnormal PC rhythmicity is transmitted to DCN neurons with no effect on their mean firing frequency. Long term depression at the parallel-PC synapses was altered and the intra-burst complex spike spikelets frequency was increased without modification of the mean complex spike frequency in BK-PC^−/−^ mice. We argue that the ataxia present in these conditional knockout mice could be explained by rhythmic disruptions transmitted from mutant PC to DCN but not by rate code modification only. This suggests a neuronal mechanism for ataxia with possible implications for human disease.

## Introduction

BK channels are large-conductance voltage and Ca^2+^-activated K^+^ channels acting as important signals modulators in many types of neurons^1–3^. They are activated by the conjunction of membrane depolarization and intracellular Ca^2+^ concentration ([Ca^2+^]_i_), and yield strong K^+^ currents. These currents play different roles ranging from regulation of transmitter release^4^ to shaping of dendritic Ca^2+^ spikes^5^ and modulation of action potential repolarization^6,7^. The functional link between BK channels activation and elevation in [Ca^2+^]_i_ is reinforced by a macromolecular complex formed by the association of BK and voltage-gated Ca^2+^ channels (Cav). This requires a rise in [Ca^2+^]_i_^3^, which can be provided by activated N-methyl-D aspartate (NMDA) receptors^8^.

In cerebellar Purkinje cells (PC), BK channels are highly expressed in the soma and dendrites^9,10^. They are activated by Ca^2+^ provided by P/Q-type Cav^11^. In turn, BK channels activation counteracts the inward Ca^2+^ current effect^5^. This mechanism plays an important role with respect to climbing fiber (CF) response characterized by a burst of three to five Na^+^ spikes and a dendritic Ca^2+^ spike in the target PC^12–15^. The CF triggers one of the most important Ca^2+^ entrance in a brain cell^16^. In accordance with Ca^2+^ influx control exerted by the BK channels, Chen *et al*.^17^ demonstrated that the CF–evoked dendritic Ca^2+^ transients had larger amplitude in PC-BK^−/−^ mice. Consequently, the BK channel could play a key role in PC by limiting Ca^2+^ entrance during CF input^18^. This tight regulation underlies crucial Ca^2+^ functions in neuronal excitability and plasticity. This calls for the investigation of long term depression (LTD) at the parallel fiber-PC-BK^−/−^ synapse for which the calcium signaling is central^19^. In humans, the recent description^20^ of cerebellar ataxia associated with *KCNMA1* gene mutation, presumably causing BK channel loss of function, reinforces the importance of the study of PC-BK^−/−^ mice.

In alert animals BK channels deletion in the cerebellum led to the emergence of beta oscillation phase-locked with ultra-rhythmic PC and Golgi cells^21^. As the sole output of the cerebellar cortex, the PC exhibits a double rhythmicity which is associated with ataxia^21^. The functional link between ataxia and the deletion of BK channels in the cerebellum is strengthened by the fact that we could reproduce the PC firing pattern (including abnormal rhythmicity) and the ataxic behavior by micro-injection of BK channels blocker (paxillin) in the cerebellum of alert WT mice^21^. Nevertheless, the precise role played by the PC-BK channels has not been elucidated in the alert state. In this perspective, the generation of a mouse line with a PC-specific deletion of BK channels (PC-BK^−/−^) offers an ideal model for studying the PC-BK channels’ role in cerebellar physiology. Interestingly, Chen *et al*. (2010)^17^ demonstrated the presence of an ataxic behavior in those mutant mice (PC-BK^−/−^). Thus, there is a physiological link between PC-BK channels deletion and ataxia. However, cerebellar rhythmicity has not been studied to date in this model. If there is a functional link between increased rhythmicity and ataxia^21^, (1) selective suppression of PC-BK channels should produce abnormal rhythmicity in the cerebellar cortex and (2) this abnormal rhythm should be transmitted to the deep cerebellar nuclei (DCN).

The role of the DCN is crucial as they are not only the final stage of the cerebellar circuit receiving PC inhibitory modulation but also an integrating network combining this inhibition with direct mossy fiber and CF excitatory inputs, and intrinsic properties^22–30^. Therefore, studying DCN modulation is essential to understand the pathophysiology of cerebellar ataxia^31^. Based on rate code modulation only, a decrease in PC discharge frequency should logically increase DCN output frequency toward the premotor target and also increase inhibition of the IO, reducing CF output frequency, which would in turn destabilize the PC networking, as demonstrated in anesthetized animal^17^. However, the mean firing rate of the different components of the olivo-cerebellar system does not describe the dynamics of the systems accurately. As demonstrated by Person and Raman^30^ the mode of firing of PC and their synchronicity strongly influence DCN firing in a way that the firing rate code only cannot predict. An increase of PC synchronization (without any firing rate modification) may ‘paradoxically’ lead to an increase of the DCN firing rate. Moreover, the temporal pattern of PC firing is highly dependent on alertness^32–34^.

To address these issues we used multiple-unit extracellular electrodes in awake mice and analyzed the rhythmicity of the PC simultaneously with the DCN neurons. We showed in alert mice that selective deletion of the PC-BK channels –as convincingly demonstrated in supplementary Figure S1^17^– induces increased simple spikes (SS) rhythmicity of the PC, increased frequency of the complex spike (CS) spikelets, and alters LTD at the parallel-PC synapse. We also demonstrate that an abnormal PC rhythmicity is transmitted to the DCN neurons. Rather than rate code modification, rhythm disruptions transmitted from PC to DCN is probably underlying the ataxic phenotype. Therefore, despite strong recurrence in the cerebellum, suppression of a single membrane ion channel type in PC population markedly disrupts cerebellar function. Our findings suggest a possible neuronal mechanism for cerebellar ataxia.

## Results

**Figure 1:**
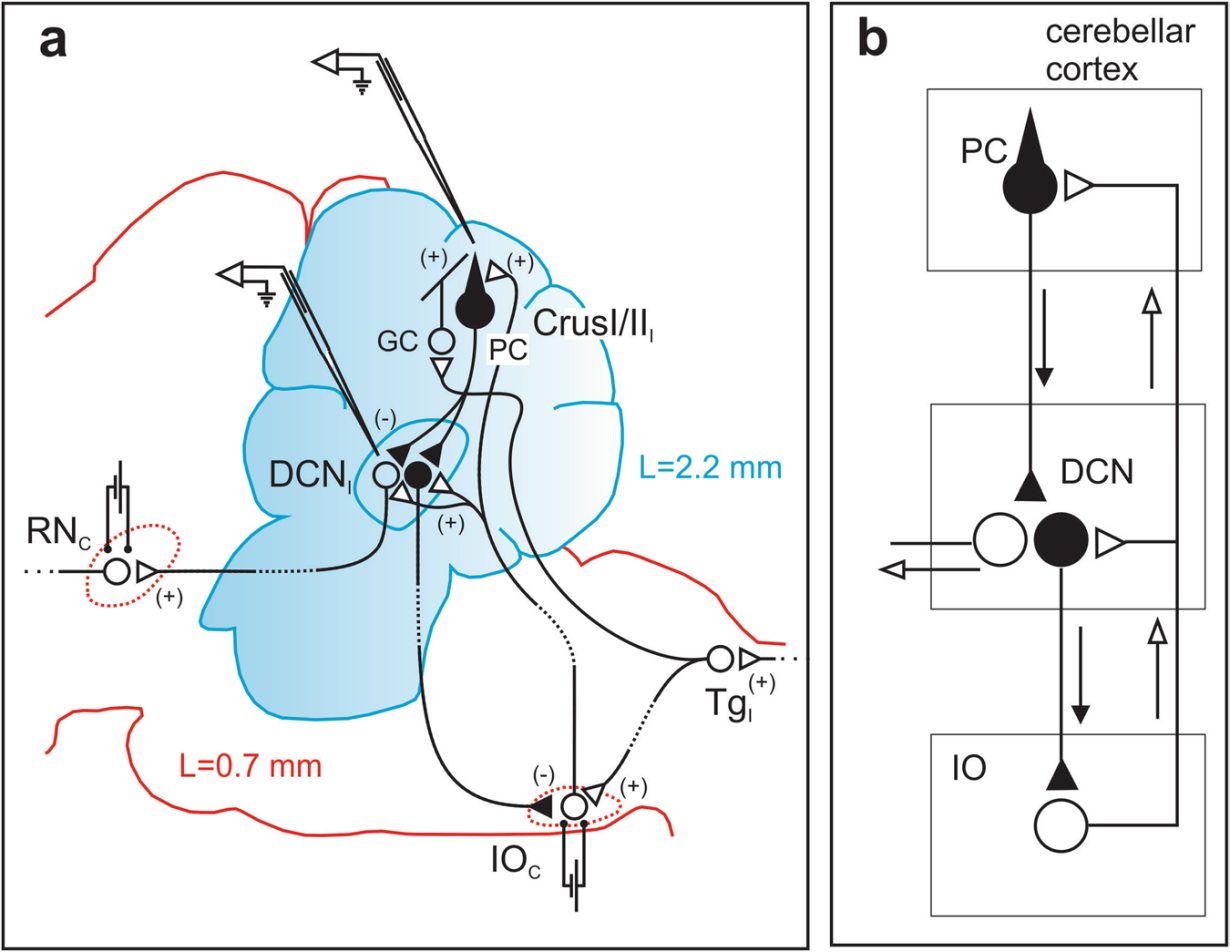
Schematic representation of the circuit involved in the present experimental paradigm. (**a**) PC of the Crus II and the DCN neurons (lateral nucleus) are recorded. Bipolar stimulation electrodes are chronically implanted in the red nucleus (magnocellular part) and in the inferior olive (dlPO dorsal lamella of the principal olive) of the contralateral side (RNc and IOc, respectively). The cortico-nuclear inhibitory projection of the PC to the DCN and the nucleo-olivary pathway are represented in black, the loop is closed by the climbing fiber (CF) projection (open symbol) from the IOc to the PC of the Crus II. The excitatory pathway from the DCN to the RNc is also represented. The pathway involved in the 8 Hz-inducing LTD paradigm is represented by the electrical stimulation of the whisker pad transmitted ipsilaterally via the trigeminal nucleus to the cerebellar mossy fiber (MF) producing the final excitatory input of the parallel fiber to the Crus II PC. (**b**) Simplified circuit of the olivocerebellar pathway presenting the 3 classical elements (the cerebellar cortex, the deep cerebellar nucleus and the inferior olive). The vertical arrows represent the signal circulation inside of the loop and the horizontal arrow the final output elaborated by the DCN neurons.

Using multi-electrode extracellular recording techniques in the alert animal, we recorded the firing behavior of the PC of Crus IIa and the DCN neurons identified from antidromic activation from the red nucleus and their electrical activation from the inferior olive (IO) (see Methods) (Fig. 1).

### Firing behavior of PC-BK^−/−^ PC in alert state

**Figure 2:**
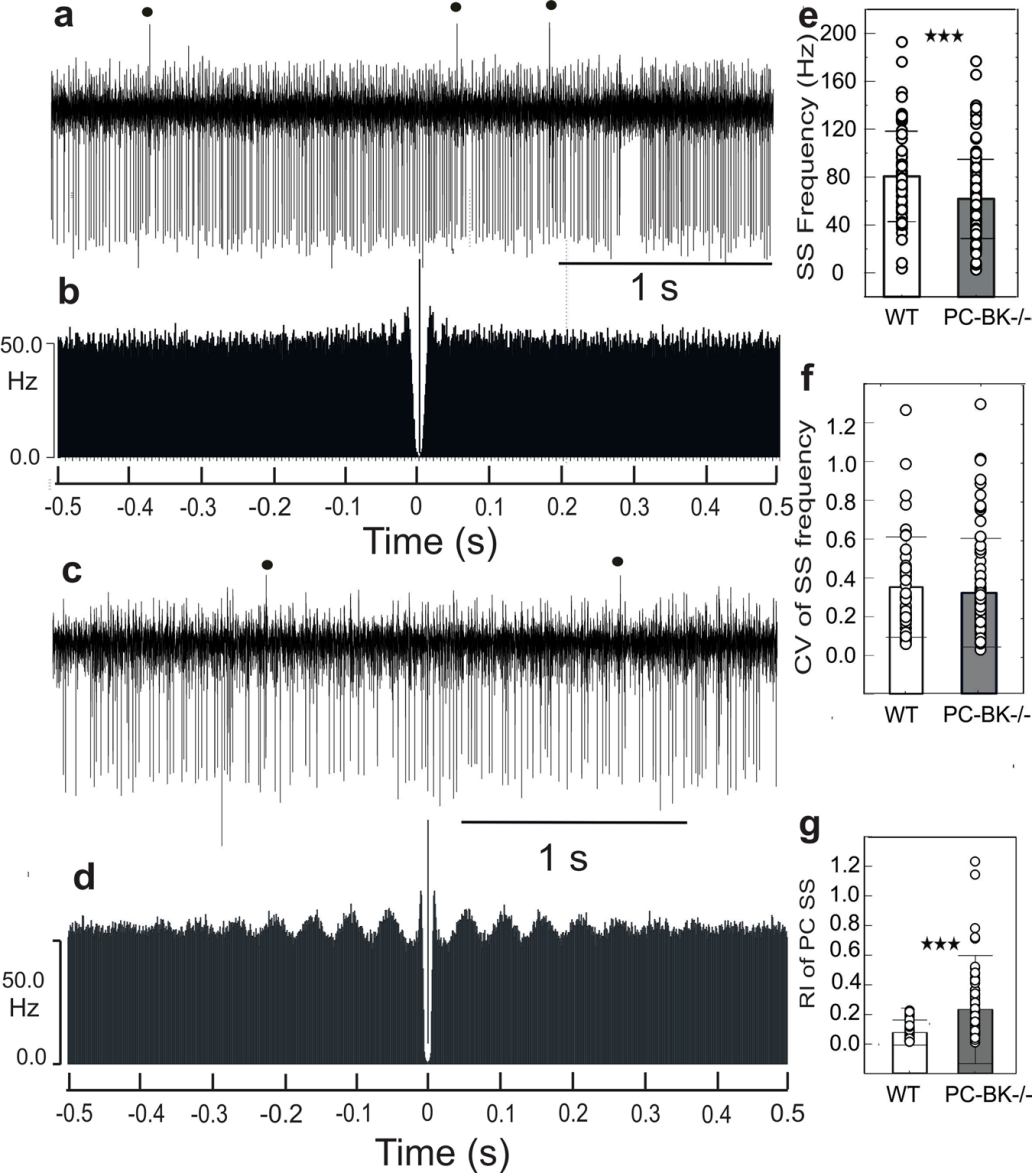
Rhythmic alteration of the SS firing behavior in PC-BK^−/−^ mice. (**a**) PC firing behavior in one WT mouse. CS are marked by a black dot. (**b**) Autocorrelogram of the SS firing illustrated in (**a**). (**c**) PC firing behavior of a representative PC-BK^−/−^ mouse. (**d**) Autocorrelogram of the SS firing illustrated in **c**. Note the numerous side peaks corresponding to beta oscillation (19.3 Hz).(**e**) Histogram (**e**,**f,g**) Box and whisker plots (mean ± SD) of the SS frequency, the CV of the SS frequency and the RI of the SS PC, respectively. The PC-BK^−/−^ mice are represented with the grey bars and the WT with the white bars. (***, for P < 0.001).

A total of 194 PC (127 in PC-BK^−/−^ and 67 in WT) were recorded and analyzed in 33 mice (18 PC-BK^−/−^ and 15 WT mice). SS firing may be considered as an important criterion reflecting the functional interaction between intrinsic PC pacemaker properties and the influence of afferent- and network-related input. As previously observed in alert total BK channels–deficient mice^21^ and in anesthetized PC-BK^−/− ^^17^, SS firing frequency was lower in PC-BK^−/−^ (61.7 ± 33.0 Hz; n = 127 cells and 18 mice) than in WT mice (80.5 ± 37.7 Hz; n = 67 and 15 mice; P < 0.0004) (Fig. 2e). There was no difference in the coefficient of variation (CV) (0.32 ± 0.27 in PC-BK^−/−^, versus 0.35 ± 0.25 in WT, P = 0.44) (Fig. 2f).

In order to verify that the selective suppression of the BK channels in the PC is able to produce increased PC rhythmicity, the rhythm index (RI) was measured during spontaneous PC activity. This analysis demonstrated that the RI considerably increased in the PC-BK^−/−^ mice reaching a mean value of 0.22 ± 0.36 (n = 127 cells) contrasting with 0.06 ± 0.08 (n = 67 cells) in WT; P < 0.0005 (Fig. 2g). Figure 2 illustrates the rhythmic alteration of the SS firing behavior in PC-BK^−/−^ compared to WT mice. A clear oscillation in the beta band (19.3 Hz in the present case) emerges with numerous side peaks in the PC-BK^−/−^ PC (Fig. 2c,d) in contrast to WT (Fig. 2a,b).

**Figure 3:**
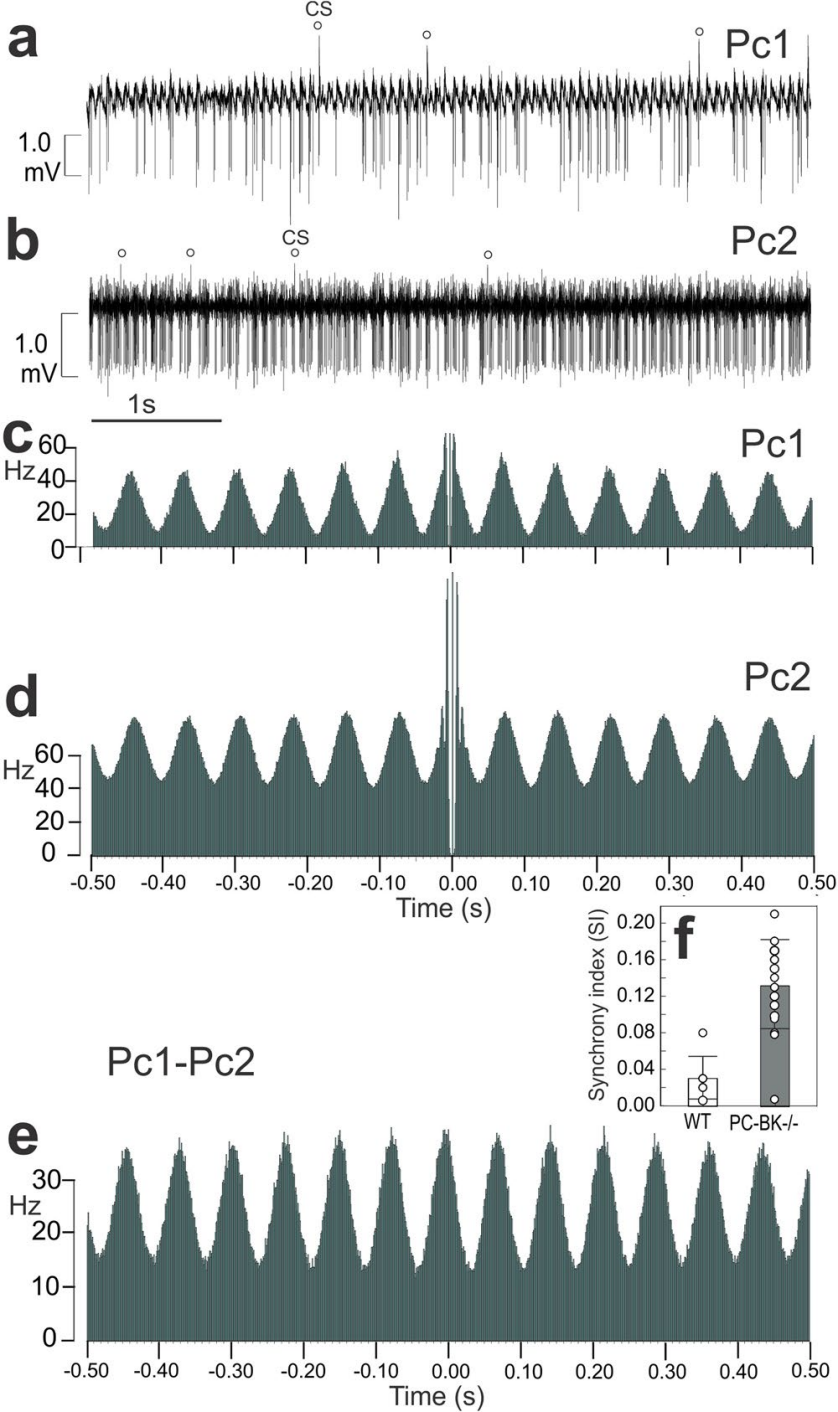
Synchronization of the SS firing along the PF beam in PC-BK^−/−^ mice. (**a**,**b**) Firing patterns of two PC (Pc1, Pc2) distant from 250 µm along the parallel fiber beam. CS occurrence is marked by an open circle. (**c**,**d**) Autocorrelograms of the SS firing of the Pc1 and Pc2, respectively. These autocorrelograms show a double rhythmicity related to the fast intraburst frequency (~ 200 Hz, center of the autocorrelogram) and the periodic bursting pattern in the beta range (~14 Hz) presenting numerous side peaks keeping the same amplitude along time. (**e**) Crosscorrelogram of the SS firing of the same PC illustrated in (**c**,**d**). This analysis was performed during a continuous time recording of 7 minutes indicating the robustness of the beta oscillation. (**f**) Box and whisker plots (mean ± SD) of the synchrony index (SI) measured in 18 PC pairs of PC-BK^−/−^ and 7 PC pairs of WT mice. (***, for P < 0.001).

Among the 127 PC recorded in PC-BK^−/−^ mice, 61 (48%) showed a double rhythmicity with a periodic bursting pattern in the beta range (17.6 ± 5.9 Hz) and fast intraburst frequency (190 ± 57.2 Hz) (Fig. 3a–d). 55 PC (43.3%) showed tonic firing with fast rhythmicity (140.5 ± 48.5 Hz). The remaining 11 cells (8.7%) showed a similar pattern to the majority (61%) of PC recorded in WT animals, i.e. SS firing without any consistent rhythmicity (RI ≤ 0.05), as illustrated by flat autocorrelogram (Fig. 2b). Paired recordings showed that the SS firing of the PC-BK^−/−^ was synchronous along the parallel fiber beam (Fig. 3). Figure 3 illustrates this highly rhythmic SS firing pattern in two PC distant from each other by 250 µm. Both autocorrelograms show the double rhythmicity, the slow one peaking at about 14 Hz and the fast one showing two fast side peaks in the second PC (Fig. 3c,d). In addition, the crosscorrelogram of these two cells shows the same beta rhythmicity (~14 Hz) indicating in-phase synchrony of these abnormal synchronized firing patterns. This in-phase synchronicity was quantified by means of the synchrony index (SI), showing a highly significant difference (P < 0.00002) between PC-BK^−/−^ (0.13 of ±0.04, n = 18) and PC-WT pairs (0.03 ± 0.02, n = 7).

**Figure 4:**
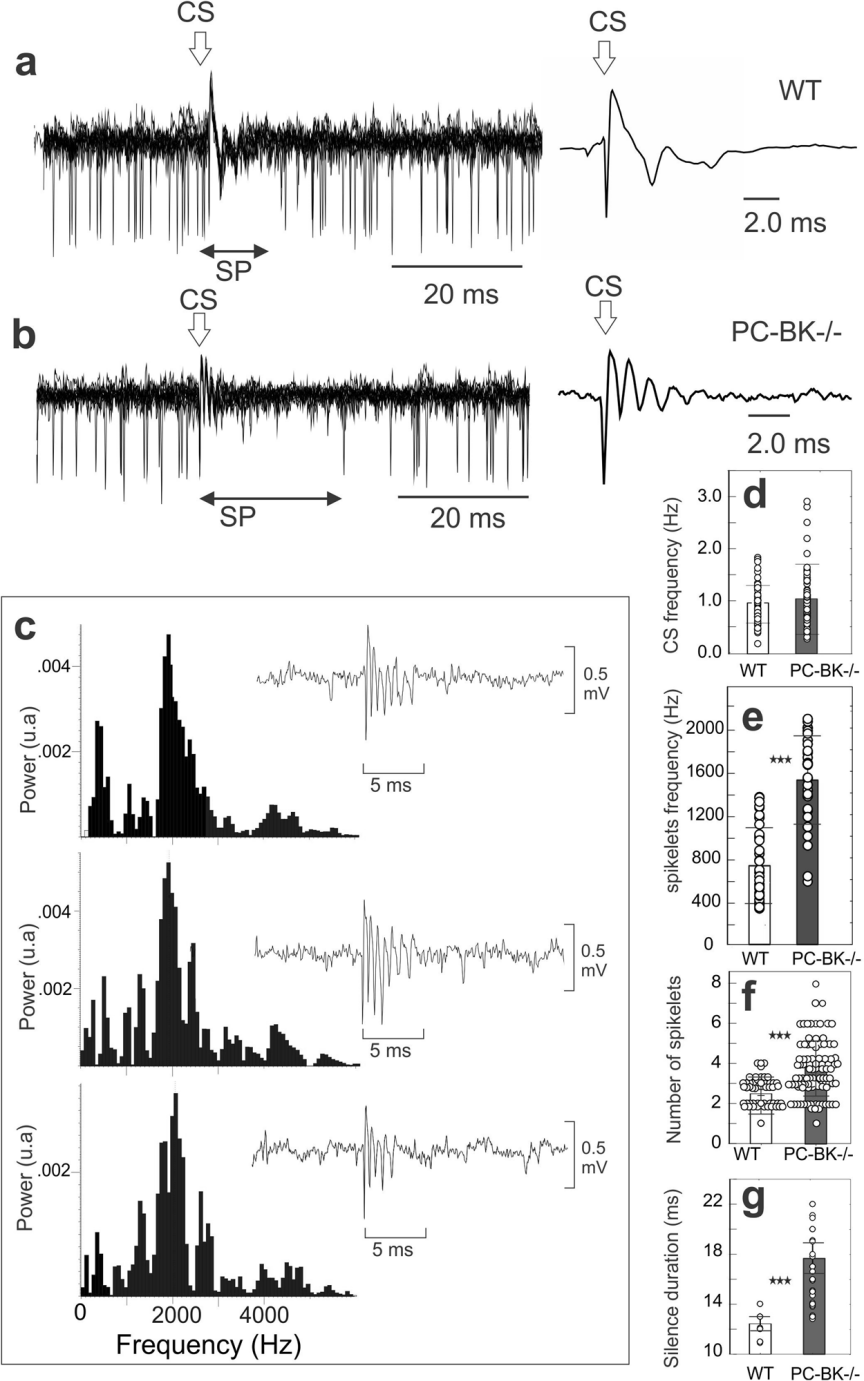
Increase of the intraburst frequency of the CS spikelets and SS silent period in PC-BK^−/−^ mice. Left, superimposition of CS occurrence (n = 10)(marked by an arrow) triggering the silent period (SP) of the SS firing in an WT (**a**) and a PC-BK^−/−^ mice (**b**). Right, averaged trace (n = 10) of the corresponding CS. Note the increased number of spikelets in the PC-BK^−/−^ mouse. (**c**) FFT analysis performed around 3 isolated and consecutives CS (illustrated in the corresponding inset) highlighting the very high intraburst frequency of the CS in the PC-BK^−/−^ mouse. =of the CS frequency (**d**), the intraburst frequency of the CS spikelets (**e**), the number of spikelets (**f**) and the SS silence evoked by the CS (**g**). The PC-BK^−/−^ mice are represented with the grey and the WT with the white bars. (***, for P < 0.001).

Figure 4a–g illustrates the alteration of the CS behavior: CS frequency was similar in PC-BK^−/−^ and WT mice (1.02 ± 0.67 Hz, n = 127 cells in PC-BK^−/−^, 18 mice versus 0.93 ± 0.36 in WT, n = 67 cells, 15 mice; P = 0.30) (Fig. 4d). In contrast, intraburst of spikelets frequency was significantly higher in PC-BK^−/−^ (1517.7 ± 406.5 Hz (n = 34 cells) than in WT mice (722.1 ± 343.3 Hz, n = 41 cells; P < 0.0000) (Fig. 4b,e). FFT analysis performed on isolated CS confirmed this increase in spikelets frequency in the mutant mice (Fig. 4c). Accordingly, the number of spikelets was higher in PC-BK^−/−^ mice (3.7 ± 1.3 n = 98 cells) (Fig. 4b,f) than in WT mice (2.3 ± 0.9, n = 67 cells; P < 0.000001) (Fig. 4a,f) while CS duration was similar (6.8 ± 3.3 ms, n = 64 cells in PC-BK^−/−^ versus 7.1 ± 1.7 ms, n = 62 cells in WT; P = 0.49). The silencing of SS firing evoked by the CS was longer in PC-BK^−/−^ mice (17.6 ± 11.0, n = 79 cells) (Fig. 4b,g) than in WT (12.4 ± 4.5 n = 67 cells; P < 0.0003) (Fig. 4a,g).

### Firing behavior of the DCN neurons in alert state

**Figure 5:**
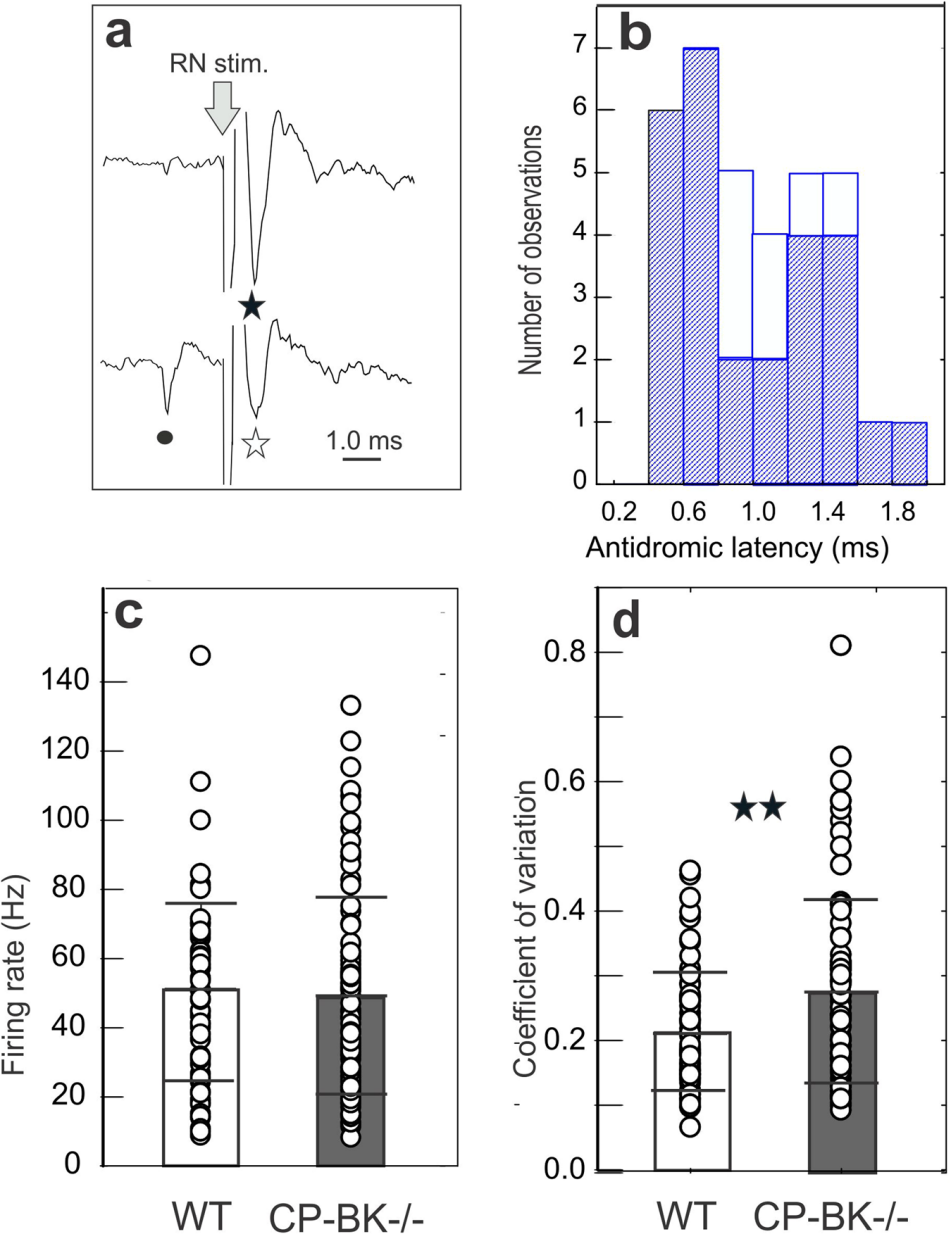
Identification of the DCN neurons and firing rate properties. (**a**) Example of antidromic activation (black asterisk) of a DCN neuron from stimulation of the red nucleus (RN stim., arrow). Note the collision (white asterisk) between the spontaneous spike of the DCN neuron (black point) and the antidromic spike. (**b**) Histogram of the antidromic latency of the DCN neurons activated from the red nucleus in PC-BK^−/−^ (gray) and in WT mice (white). (**c**,**d**) Box and whisker plots (means ± SD) of the DCN neurons firing rate, and coefficient of variation, respectively. The PC-BK^−/−^ mice are represented in black bar and the WT in white bar. (**, for P < 0.01).

A total of 154 DCN neurons (90 in PC-BK^−/−^ and 64 in WT) were recorded and analyzed in 33 mice (18 PC-BK^−/−^ and 15 WT mice). In order to facilitate online recognition of the DCN among these neurons, 41 (27 in PC-BK^−/−^, 14 in WT) were antidromically activated from the red nucleus (Fig. 5a) at a mean latency of 1.11 ± 0.40 ms, which was not different for the two groups of mice (P = 0.18) (Fig. 5b). Figure 5a illustrates the antidromic activation of one DCN neuron from the red nucleus and a spontaneous collision with an orthodromic spike occurring just before the stimulation.

**Figure 6:**
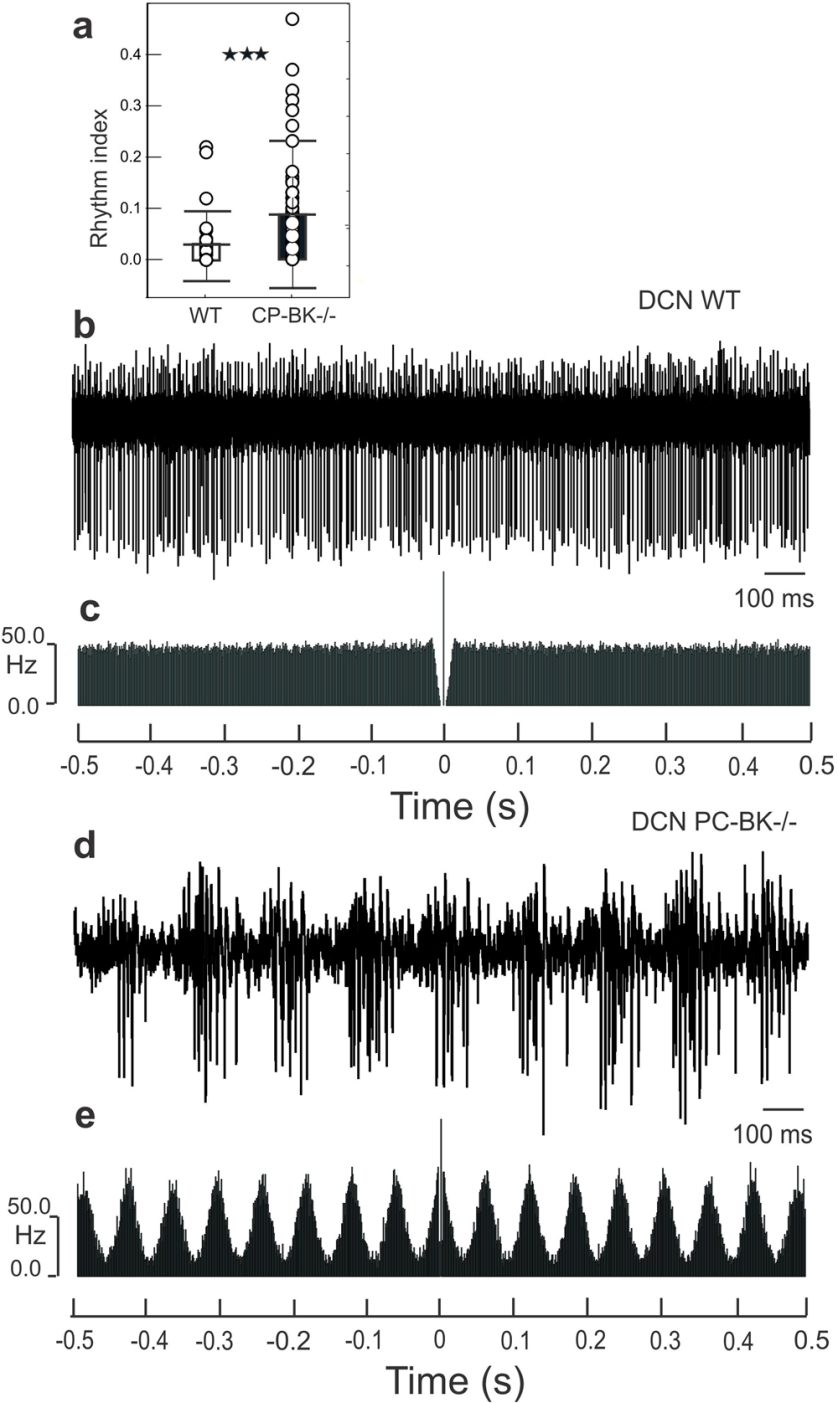
Rhythmic alteration of the DCN neurons firing behavior in PC-BK^−/−^ mice. (**a**) Box and whisker plots (means ± SD) of the rhythm index (RI) of the DCN neurons. The PC-BK^−/−^ mice are represented with the black and the WT with the white bars. (**b**) Example of firing discharge of a DCN neuron recorded in a WT mouse. (**c**) Autocorrelogram of the firing illustrated in (**b**). (**d**) Example of firing discharge of a DCN neuron recorded in a PC-BK^−/−^ mouse. (**e**) Autocorrelogram of the firing illustrated in (**d**). Note the bursting behavior and the constant beta oscillatory profile recorded in the DCN of PC-BK^−/−^ mouse in contrast to the tonic firing and the flat autocorrelogram in WT mice. (***, for P < 0.001).

Although the logical consequence of the SS firing decrease in the mutant would be to induce disinhibition of the DCN neurons, the mean frequency (48.4 ± 28.5 Hz in the PC-BK^−/−^ (n = 90) versus 49.7 ± 25.6 Hz in WT, n = 64) (Fig. 5c) was not different (P = 0.76) but the CV was increased in the PC-BK^−/−^ (0.27 ± 0.14) with respect to the WT (0.21 ± 0.09)(P < 0.003)(Fig. 5d). As for the PC, the DCN neurons showed a significant RI increase reaching a mean value of 0.08 ± 0.14 (n = 90) in the PC-BK^−/−^ mice in place of 0.02 ± 0.06 (n = 64) in the WT; P < 0.005 (Fig. 6a). Figure 6 illustrates the marked change in the rhythmic behavior of one representative DCN neuron in a PC-BK^−/−^ mouse showing beta bursting (Fig. 6d) that gives rise to a constant oscillatory profile as shown in an autocorrelogram (Fig. 6e) instead of the tonic firing (Fig. 6b) and flat profile (Fig. 6c) seen in the WT DCN neuron. The beta oscillatory profile has been identified in 41% of the DCN neurons of the PC-BK^−/−^ mice, reaching a mean value of 16.9 ± 5.7 Hz, n = 37. Interestingly, the mean oscillatory profile of these DCN neurons was not statistically different (P = 0.58) from the one recorded in the PC of the same mutant mice.

When the DCN neurons antidromically activated by a stimulation of the red nucleus were separately analyzed from the DCN neurons that were not identified as providing input to the red nucleus, the same differences in firing parameters (frequency rate, CV and RI) between the mutant and WT mice were found. The mean total duration of the DCN neuron spikes (n = 139) was 6.2 ± 2.8 ms and the depolarization phase was 0.36 ± 0.19 ms. These spike parameters were not different in mutant and WT mice (P = 0.41 and P = 0.66, respectively).

### Effects of IO stimulation on DCN neurons

**Figure 7:**
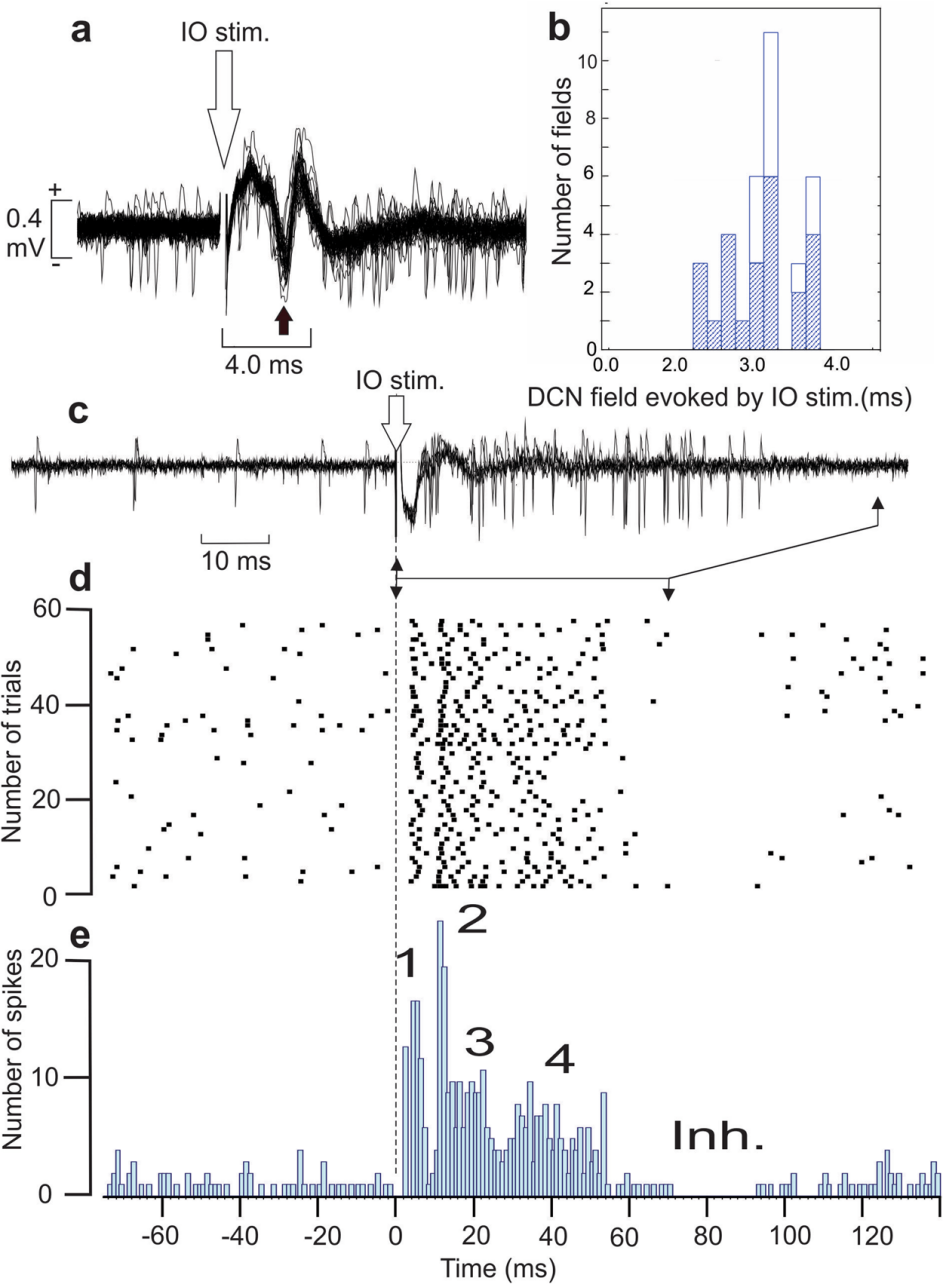
Activation of the DCN neurons by the stimulation of the inferior olive nucleus. (**a**) Superimposition of the firing discharge of a DCN neuron during the stimulation (n = 54) of the inferior olive in a WT mouse. Note that the spikes are superimposed on a negative field potential occurring at about 3 ms. (**b**) Latency histogram of the negative peak recorded in WT (white areas) and in PC-BK^−/−^ mice (gray areas). (**c**) Another example of DCN neurons activated by the inferior olive stimulation (n = 5) (IO stim.) but for which the negative field is less marked than in A allowing a better identification of the early spike responses. (**d**) Raster histogram of the DCN neuron illustrated in (**c**). (**e**) Histogram of the spike evoked responses produce by the stimulation of the inferior olive showing 4 successive activation sequences (1, 2, 3, 4) followed by a delayed inhibition (inh.).

In order to verify the functional integrity of the IO action on the DCN, the effect of IO stimulation was analyzed in 48 DCN neurons (28 in PC-BK^−/−^ and 20 in WT). This stimulation evoked a negative field potential (Fig. 7a) at the latency of 3.2 ± 0.5 ms in the majority of these neurons (n = 35) and no difference was found between the mutant and WT mice (P = 0.12) (Fig. 7b). Although the placement accuracy of the stimulating electrode (online) in the IO was confirmed by the fact that they induced a specific negative field in the PC layer (as demonstrated in the ferret)^35^ and followed by evoked CS at a latency ranging from 10 to 20 ms and resembling to the spontaneous one (Fig. S1), we never succeeded in recording antidromic activation of DCN neurons from the IO stimulation.

IO stimulation also induced an increase in DCN neurons firing rate expressed in different peaks in the stimulus histogram (Fig. 7c–e). All these sequences (excitatory and inhibitory) were not different in the mutant with respect to the WT mice. The first peak occurred at the latency of 5.9 ± 1.3 ms in PC-BK^−/−^, (n = 24) versus 6.2 ± 1.8 ms in WT, (n = 20), (P = 0.45); the second one at 12.7 ± 2.6 ms, in PC-BK^−/−^, (n = 17) versus 11.2 ± 2.5 ms in WT (n = 16), (P = 0.10); the third at 20.7 ± 1.2 ms in PC-BK^−/−^, (n = 8) versus 25.5 ± 2.5 ms in WT (n = 12), (P = 0.079); and the fourth at 50.0 ± 16.1 ms in PC-BK^−/−^, (n = 10) versus 46.3 ± 4.4 ms in WT (n = 6), (P = 0.79). This excitatory sequence was ended by a period of inhibition of variable duration centered on 110.7 ± 31.9 ms at the onset latency of 82.5 ± 42.3 ms in PC-BK^−/−^, (n = 10) versus 76.0 ± 24.1 ms in WT (n = 6), (P = 0. 79). All these sequences (excitatory and inhibitory) were not different between the knockout and the WT mice.

### Rhythmic communication between PC and DCN in PC-BK^−/−^

**Figure 8:**
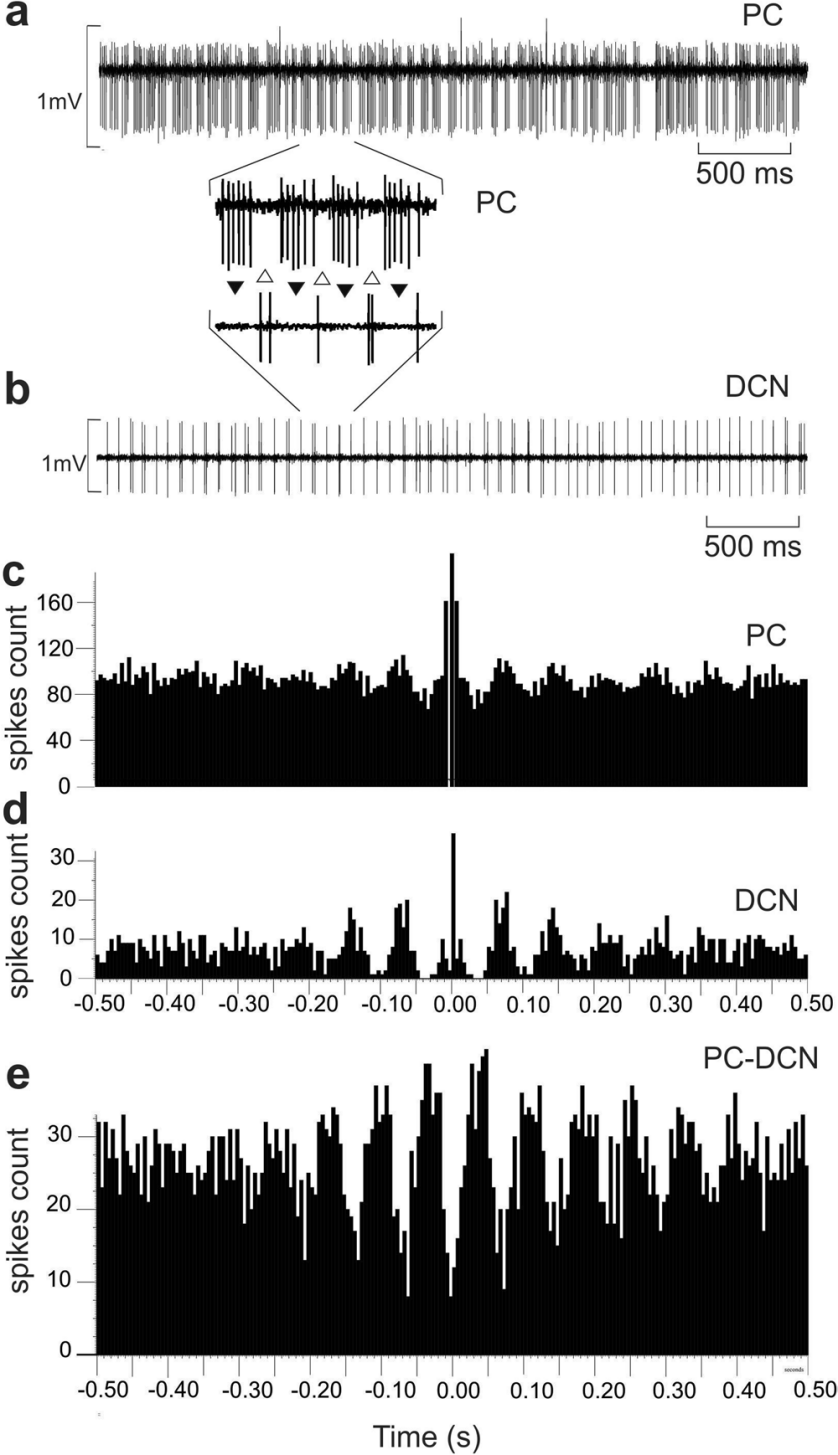
Beta rhythm transmission from PC output to the DCN neurons. Simultaneous recordings of one PC and one DCN neuron in a PC-BK^−/−^ mouse. (**a**) PC firing rate. (**b**) DCN neuron firing rate. (between **a** and **b**) magnification of 4 bursts containing 5 to 6 SS spikes corresponding to the absence of firing of the DCN neuron. The reciprocity of firing is indexed by the alternation of black and white triangles. (**c**) Autocorrelogram of the SS firing of the PC illustrated in (**a)**. (**d**) Autocorrelogram of the DCN neuron illustrate in (**b**). (**e**) Crosscorrelogram of the PC SS firing and the DCN neuron firing. Note the presence of numerous side peaks in the respective autocorrelograms (**c** and **d**) and the out-of-phase functional correlation in (**e)**.

**Figure 9:**
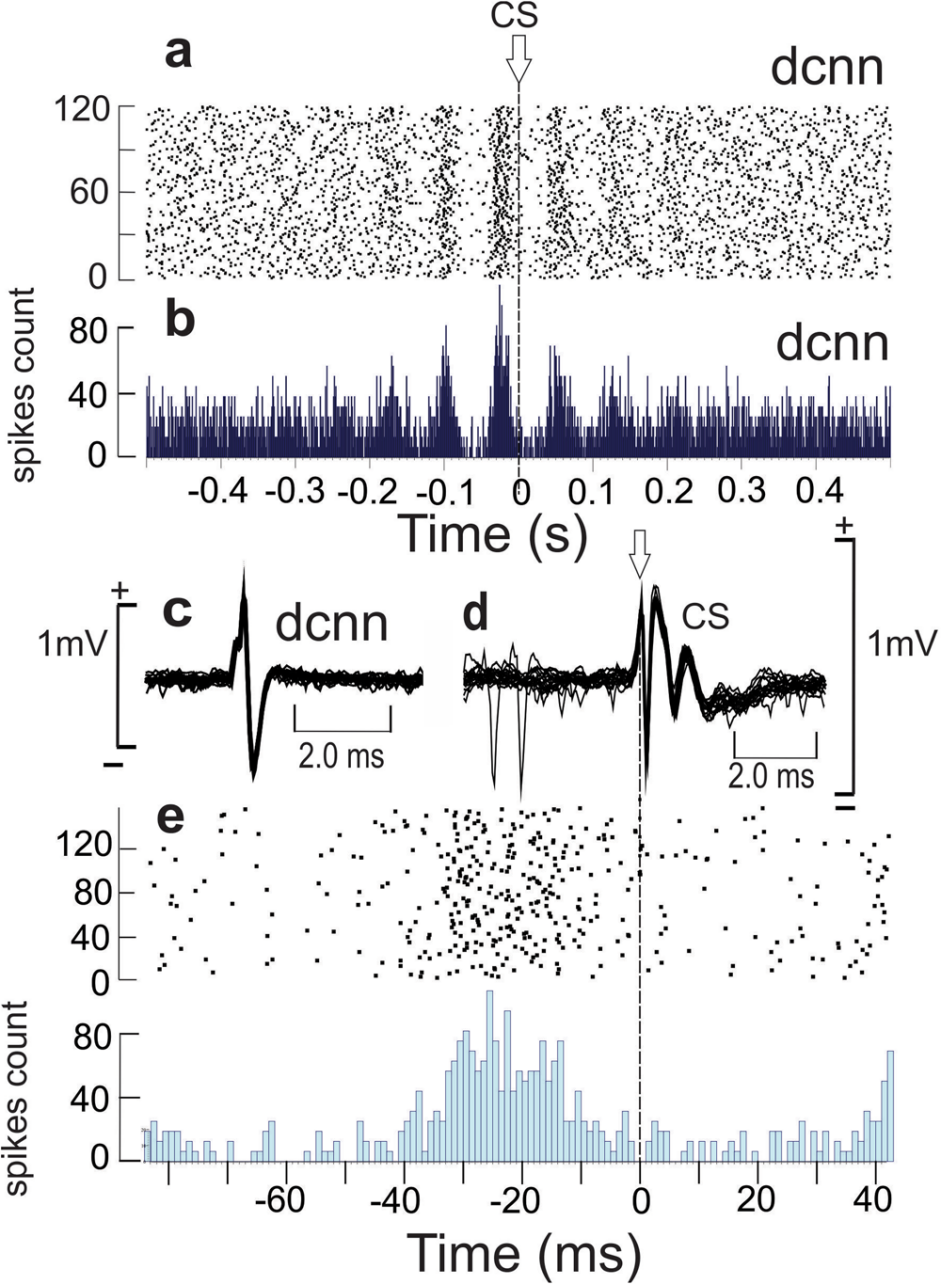
Functional relationship between the DCN neuron activity and the CS firing in PC-BK^−/−^ mouse. (**a**) Raster histogram of a DCN neuron triggered by the CS of the simultaneous recorded PC. (**b**) Correlogram corresponding to the DCN neuron firing illustrated in (**a**). (**c**) Superimposition of the DCN neuron spikes (n = 18). (**d**) Superimposition of the CS of the PC firing. Note the presence of two SS before the superimposed CS (n = 16). (**e**) Time expansion of the central part of the raster and correlogram illustrated in (**a**,**b**) showing that the peak of the DCN neuron firing occurs about 26 ms before the CS occurrence.

In some exceptional recordings performed in PC-BK^−/−^ mice, we were able to simultaneously record pairs of PC and DCN neurons. Figure 8 illustrates one PC showing the double rhythmic pattern (Fig. 8a) and one DCN neuron presenting the same beta rhythmicity at about 15.3 Hz (Fig. 8b) in an exact out-of-phase relationship (Fig. 8d). It is technically difficult to keep one PC and one identified DCN neuron in alert animal for quality recording, but the existence of such functional pairs (n = 6) may contribute to a better understanding of the olivo-cerebellar network. The magnification part of Fig. 8 illustrates a series of 5 to 6 SS spikes followed by one or 2 DCN spikes (while the PC were silent). The autocorrelograms of the PC (Fig. 8c) and DCN neuron firing (Fig. 8d) confirm the presence of the same beta rhythm. The crosscorrelogram between these two units demonstrated their functional out-of-phase correlation (Fig. 8e). This suggests a transmission of the abnormal beta rhythm by PC inhibition to the DCN neuron. In addition, the analysis of the relationship inside the PC-DCN pairs between the firing activity of the DCN neuron and the CS activity of the related PC demonstrated a phase-locked of the DCN firing to the generation of the CS of the original PC (Fig. 9). This corroborates the existence of the closed loop between the DCN to the PC via the IO. When the DCN firing is aligned with the CS occurrence a clear beta oscillation was recorded in the DCN firing. The major peak of DCN discharge preceded the CS by 29.2 ± 3.9 ms (n = 6) (26 ms in Fig. 9).

### 8Hz LTD-inducing paradigm

**Figure 10:**
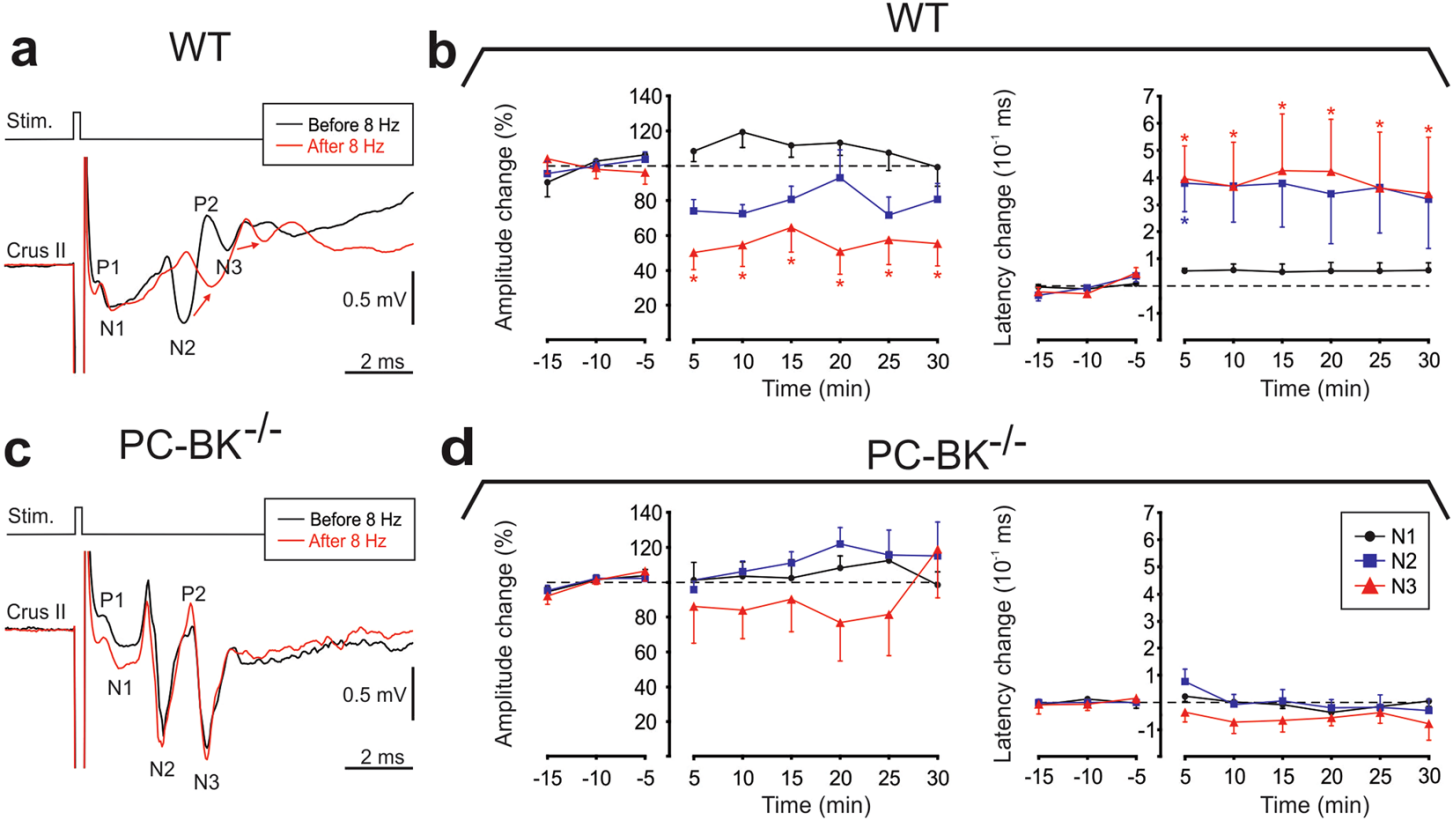
Absence of evoked plasticity (LTD) in PC-BK^−/−^ mice. (**a**) Superimposition of the local field potential components (P1, N1, N2, P2, N3) evoked by single whisker stimulation before (black traces) and after 8 Hz LTD-conditioning stimulation (red traces) (pulse signal, Stim.); recorded in Crus IIa in WT mouse. Note the decrease of N2 and N3 amplitude and the related latency shift of these components after 8 Hz LTD-conditioning stimulation. (**b**) Changes in amplitude and latency of evoked local field potential components N1 (black dots), N2 (blue squares) and N3 (red triangles), 15 min before and 30 min after the 8 Hz LTD-conditioning stimulation in WT mice. (**c**) Same type of local field potential superimposition that in (**a)** but recorded in a PC-BK^−/−^ mouse. Note the absence of amplitude decrease and latency shift of N2 and N3. (**d**) Changes in amplitude and latency of evoked local field potential components as in (**b**) but for the PC-BK^−/−^ mice. Data points are expressed as mean ± SEM. Significant differences (p < 0.05) from control (pre-stimulation period) are indicated with asterisks.

In order to highlight the potential role of PC-BK channels in the sensory-stimulation plasticity observed in the cerebellar cortex of WT alert mice^36^, we investigated the local field potentials (LFP) plasticity recorded in the Crus I or Crus II area, evoked by electrical stimulation of the whisker pad. Ten recordings were performed for each group and were obtained in 6 mutant and 5 WT mice. This stimulation evoked three early negative waves, designated as N1, N2 and N3, peaking at 1.8 ± 0.4 ms, 3.3 ± 0.3 ms, and 4.5 ± 0.2 ms (n = 15), respectively, after stimulus onset. The mean peak-to-peak amplitude of these components corresponded to 0.19 ± 0.15 mV, 0.43 ± 0.18 mV, and 0.48 ± 0.19 mV, respectively (mean ± SD; n = 15). The short latency and small amplitude of N1 correspond to the classical P1–N1 presynaptic input component^37^ originating in the mossy fiber in the granule cell layer. N2 and N3 are related to PC activation mainly supported by the ascending axon–PC synapse and the parallel fiber-PC synapses, respectively^36^. In WT mice, we observed long-term modifications (lasting at least 30 min) after 8-Hz stimulation, characterized by a specific decrease of the N3 amplitude accompanied by an increase of the N2 and N3 latency peaks (Fig. 10) expressing an LTD effect (P < 0.05). In contrast, neither effects on N3 amplitude nor on N2, nor on N3 latency peaks occurred in the PC-BK^−/−^ (Fig. 10). These results strongly suggested that PC-BK channels play a major role in the LTD timing-plasticity.

## Discussion

Our results showed that specific ablation of BK channels in PC induced a stereotyped SS firing rhythmic alteration that was transmitted to DCN neurons, and enhanced intra-burst CS frequency. Moreover, LTD at the parallel-fiber-PC synapse was abolished. This emphasizes the importance of the olivo-cerebellar loop in the transmission between the cerebellar cortex and the DCN, and the PC-BK channels role in this network and cortical plasticity. These new findings contribute to a better understanding of the physiological link between specific deletion of a single type of K^+^ channels in a single cell population in the cerebellar cortex and cerebellar ataxia.

SS firing reduction similar to what we found in alert mice was previously observed in anesthetized preparation^17^. A causal role for decreased SS firing in the emergence of ataxia was originally hypothesized when decreased SS rate induced DCN neurons disinhibition, which in turn enhanced IO inhibition^17^. Following this IO inhibition, reduced CS frequency is expected, and was actually observed in anesthetized preparation^17^. However, our results in alert PC-BK^−/−^ mice did not reproduce these findings, suggesting that reduced CS frequency *per se* is not a pathophysiological mechanism and that they may reflect an effect from anesthesia. Another major difference with the anesthetized preparation is the absence of increase in mean discharge frequency of DCN neurons in alert PC-BK^−/−^ mice. More generally, DCN neurons discharge frequency has been reported to be much lower compared to our findings in alert mice (e.g. 10.5 ± 2.3 Hz in WT mice and 14.7 ± 1.9 Hz in PC-BK^−/−^ mice^17^, or 18.0 ± 13.1 Hz in L7-ChR2-eYFP mice^38^, versus 49.7 ± 25.6 Hz in WT and 48.4 ± 28.5 Hz in the PC-BK^−/−^ mice in the present alert condition). The absence of effect of the PC-BK deletion on DCN mean firing rate does not mean that alteration of PC output has no effect on DCN neurons. Another important parameter is the regularity of the PC, represented by the CV. In agreement with experimental evidences that irregularity of PC^39^ firing is strongly associated with cerebellar ataxia, we have also reported such irregularity in the PC of alert mice with BK channel deletion in the cerebellum^21^. However, this PC irregularity was not reproduced in the present PC-BK^−/−^ mice, but it was well expressed in the DCN neurons letting open the possibility that such irregularity in the firing output of the cerebellum could be an element sustaining ataxia.

We showed here that another parameter than the rate code is a major element in the PC-DCN transmission in alert mice. Rhythmicity of both PC and DCN neurons is dramatically increased, expressing beta oscillation. This abnormal rhythm is similar to the oscillation recorded in whole-BK mutant, as well as the one induced by BK-blocker (paxillin) in WT mice^21^. In both situations, beta oscillation was also accompanied by ataxia. Though we argued that rate code modification is insufficient to underlie ataxia but rhythmicity of different cerebellar nuclei should be taken into account, we do not exclude that other factors could also contribute to the ataxic phenotype. Indeed, enhanced excitability^31,41,40^ and firing irregularity^39^ have been shown to be important parameters in relation to ataxia.

The functional link between the rhythmic DCN output alteration and ataxia is supported by a number of electrophysiological evidences. Experiments using optogenetic stimulation in mice^42^ demonstrated that movements evoked by stimulation of the cerebellum are initially produced via a direct pathway through the red nucleus and/or the reticular formation and not by the motor cortex. This reinforces findings with electrical stimulation of the cerebellum demonstrating the powerful influence exerted by the inhibitory PC signal on descending motor pathways resulting in actual movement^43–45^. However, this feedforward control perspective is challenged by the presence of different feedback loops inside the cerebellar cortex, e.g. from the PC collaterals to the molecular interneurons, Golgi cells and other PC^44^ and from the olivo-cerebellar loop^46,47^.

We also found that this beta rhythm was functionally transmitted in an out-of-phase way. Indeed, the DCN neurons fired one or two spikes only in the absence of SS firing (at beta frequency). We showed that this rhythmic coupling is extended to the olivo-cerebellar loop as we documented the correlation between the rhythmic behavior of the DCN neurons and the CS occurrence of the related PC. This is in accordance with the recent studies of Tang *et al*.^48^ performed in anesthetized rat. They demonstrated that synchronized CS were able to induce DCN neuron inhibition which was preceded as in the present case by a short firing increase of the DCN neurons. In the same way, it was also demonstrated^38,42^ that optogenetic stimulations of a set of PC triggering restricted DCN inhibition were able to induce transient disinhibition of IO cells that project onto this specific set of PC. This optogenetic experimental strategy elegantly demonstrated in anesthetized preparation that the time between PC excitation and CS occurrence is about 64 ms (Fig. S4 in Chaumont *et al*.)^38^. In the present case, CS occurred about 29 ms after the beta firing peak of the DCN neurons. This delay is compatible with the total loop time of ~64 ms. Indeed, it takes about 20 ms for the PC to inhibit DCN neurons^38^, about 29 ms for the DCN neurons to inhibit (or disinhibit) IO neurons, the rest of the time corresponding to PC activation by the IO (~15–20 ms, Fig. S1). This looping time is also suitable for the generation of beta oscillation as we found in PC-BK^−/−^ mice.

Spontaneous recruitment of this closed cortico-nucleo-olivary loop by BK-channels deletion of PC could explain both the ataxic behavior probably sustained by the abnormal beta oscillation of the DCN output and the alteration of the LTD at the level of parallel-PC synapses. This LTD plasticity controlling the sensitivity of the excitatory input of the PC^49^ may participate in the formation and storage of internal models reproducing the sensorimotor system dynamic properties^50,51^. In this model, the cerebellar cortex integrates the current state of the sensorimotor system and internally generates motor commands to predict future action and related sensation. These predictions are transmitted via the DCN to the IO acting as comparator of the expected and ongoing action or sensation and generating the CF ‘error’ signals acting as a supervisor of cerebellar learning. This CF signal is also considered as one of the key elements of the parasagittal organization of the cerebellar module, which is subdivided in different microzones considered as the operational units of the cerebellum^52^. Acting as a supervisor on these operational units, the nucleo-olivary output requires a precise tuning^47^. Although, the preservation of the CS frequency rate in the alert PC-BK^−/−^ mice, the presence of ongoing beta rhythm coming from the DCN output probably impacts the precise tuning and accurate coupling between parallel fibers and CF input necessary for inducing LTD. As theoretically advanced by Medina and Mauk^53^, conjunction of mossy and climbing fiber input in the DCN is unable to retain memories in presence of background activities and only a plasticity rule concerning PC activity can keep a memory trace resistant to ongoing activities in the network preclude the possibility for the DCN to compensate for the PC alteration in the PC-BK^−/−^ mice. Other important elements which may explain the absence of LTD are the modification of the CS configuration and the increase of the SS silent period following the CS. It was demonstrated that in spite of the fact that the CF elicits prominent dendritic calcium spikes^14,15^ these dendritic spikes are not directly linked to the CS spikelets but well to the duration of the SS silent period triggered by the CS^54^. It was demonstrated that the silent period was correlated with the amplitude of the dendritic afterhyperpolarization^54^. The Ca^2+^ influx triggered by the CF at the dendritic level activates Ca^2+^-dependent potassium conductance^7^ inducing hyperpolarization and the modulation of the silent period in SS firing. Although, small (SK) and large (BK) conductance Ca^2+^-activated potassium channels contribute to the action potential shape and spike afterhyperpolarization, respectively^7^, the fact that the silent period was increased and not decreased in the absence of BK channels in the PC-BK^−/−^ mice (present results) and also in the whole BK^−/−^ mice and after paxilline microinjection in WT^21^ indicates that the BK channels are not implicated in this process or that their absence are compensated by another hyperpolarizing channels. It cannot be ruled out, however, that PC may compensate for the absence of BK channels by over- or under-expressing other proteins following genetic knock out.

Our finding of increased number of spikelets and SS silent period in alert PC-BK^−/−^ mice could be explained by larger amplitude CF-evoked dendritic Ca^2+^ transients as demonstrated on slice in PC-BK^−/−^ mice^17^. In addition, the increase of the number of spikelets for a same CS duration indicates that the BK channels may also regulate the production of axonal spikelets. Recent view^55^ and experimental evidences^56–58^ point out that CS configuration, namely the number of spikelets, which are directly related to the number of spikes in the CF^59^, plays a major role in PC learning. During ‘trials over trials’ learning of pursuit eye movement, Yang and Lisberger (2014, 2017)^57,58^ demonstrated that longer-duration CS related to a greater number of spikelets induced stronger learning responses than medium or short CS duration. In contrast, the SS silent period triggered by the CS did not affect learning. In the present work, increased CS spikelets number in PC-BK^−/−^ mice should favor PC learning. However, this spikelet increase was not accompanied by an increase of the CS duration but rather by an increase in spikelets intraburst frequency. Two mechanisms can be proposed to explain this: (1) as already demonstrated in slice^17^ the absence of BK disturbed PC membrane properties in such a way that a ‘conserved’ CF signal was not normally transmitted or treated by the membrane properties of the PC or (2) as it was clearly demonstrated that the phase of the subthreshold oscillation present in the IO could regulate the number of spikes in the CF burst corresponding to the CS spikelet^59^, the rhythmic alteration of the DCN inhibitory input on the IO may potentially increase the intraburst frequency of the CS. Whatever the pathophysiological pathway the alteration of the CS waveform may play a role in both the absence of LTD and the ataxia in the PC-BK^−/−^ mice.

Another important finding was the specific alteration in DCN neurons firing behavior. DCN neurons exhibit pacemaker firing properties with a mean frequency of about 25 Hz in slices^24^ and reaching higher frequencies around 90 Hz *in vivo*^22,30^. Over this ongoing activity, excitatory input from the mossy and CF collaterals and the PC inhibition are integrated to produce a tonic firing discharge that has been documented in alert animals from different species^22,23,25,29^. The specific contribution of PC has been described as convergent transduction of PC inhibitory inputs to DCN neurons, with about 11 PC projecting on to 1 DCN neuron^60,61^. It must be recognized, however, that the DCN neuron population is heterogeneous^61^. It is therefore important to identify at least one subpopulation. This can be achieved electrophysiologically by using antidromic stimulation of the red nucleus. In the alert cat DCN neurons projecting onto the red nucleus (antidromic activation at a latency of 0.76 ± 0.12 ms) have thus been characterized (firing rate from 10 to 45 Hz)^25^. Here in the alert mice we found DCN neurons antidromically activated from the red nucleus at a latency of 1.11 ± 0.40 ms. There were no differences in the firing rate of the DCN neurons projecting to the red nucleus (n = 27) compared with those which were not antidromically activated from the red nucleus (n = 81), for both groups the mean firing was about 45 Hz. The lack of statistical difference between WT and PC-BK^−/−^ mice in terms of frequency rate argued against a determining role of the DCN rate code in ataxia.

Another physiological property of the DCN operation mainly based on a timing code transmission of synchronized PC output to the DCN has recently been defended by Pearson and Raman^62^. They clearly demonstrated on slice and in anesthetized mice that the existence of a high intrinsic firing rate of the DCN neuron (91.5 ± 7.4 Hz) is differently modulated by the type of the rhythmic input of PC^30^. Asynchronous GABAergic IPSC of the PC exerts a potent inhibitory effect on the DCN neurons, but in contrast the same inhibitory input produced by synchronous PC entrains the spiking of DCN neurons in a perfect phase-locking with the rhythmic PC. This is in accordance with the present data where abnormal PC beta rhythm of the PC-BK^−/−^ mice is transmitted to the DCN neurons. The PC synchronicity observed in the present mutant may facilitate this phase-locked transmission as demonstrated by Pearson and Raman^30^ in WT mice. The present sculpting of the DCN neurons firing by the abnormal PC rhythmic pattern is accordance with the disinhibition hypothesis^50^ and with recent finding that specific inhibition of the PC by optogenetic stimulation of the basket cells in mice is able to control the timing and the kinematic properties of the cerebellar motor output via graded disinhibition of the DCN neurons^63^.

The fact that DCN response to IO stimulation was not modified in PC-BK^−/−^ mice indicates that the excitatory action of the CF collaterals on the DCN neurons is not responsible for the observed impairments, which further reinforces the importance of PC upstream signals on the DCN final output^64–66^.

Multiple conditions induce ataxia, including cerebellar ablation^26^, PC degeneration^67^ and PC firing rate reduction^9,10^. All these situations result in a decrease of GABA synaptic input on the neurons in the DCN that are targeted by PC. This has been proposed as one pathophysiological mechanism of ataxia^31^. However, in calcium-binding knockout mice^32,33,68^, and in mouse model of fetal alcohol syndrome^69^, Angelman syndrome^70^ and myotonic dystrophy^40^ ataxia has been reported in presence of SS firing increase, which is not expected to reduce the GABA synaptic input of the DCN but dramatically change the rhythmicity and the synchrony of the PC population. In addition, such SS firing increase was accompanied by abnormal fast oscillation of the local field potential disturbing the control exerted by the cerebello-olivary loop. Other, more subtle modifications of PC physiology, such as irregular PC firing, have been reported to induce ataxia^71^.

If the functional link between the absence of the classical LTD (mainly *in vitro* studies) and the behavioral learning remains controversial^72,73^, the alteration of the cerebellar LTD is often accompanied by ataxia^74–78^. Although the present LTD paradigm using 8 Hz electrical stimulation of the whiskers in alert mice is not largely used, we are confident that the N3 component is generated by the parallel fiber-PC synapse as demonstrated by collision between the activation of the granular cell axons by the peripheral stimulation and the antidromic stimulation of the parallel fiber on the cerebellar surface^36^. In addition, we have also demonstrated that both the classical LTD on slice and the 8 Hz LTD in alert mice were altered in PTPRR knockout mice^79^. This protein tyrosine phosphatase receptor type R is involved in maintaining low basal MAPK activity in PC. The same approach was also initiated in order to test the idea that cerebellar LTD may be determinant for inducing normal plasticity in the somatosensory cortex. We demonstrated that after a 8 Hz LTD inducing protocol, the cerebellar LTD accompanied by a delayed response in the WT mice was missing in Angelman syndrome mouse model and that the LTD induced in the barrel cortex following the same peripheral stimulation in WT is reversed into a LTP in the Angelman syndrome mice^80^. A mouse model for the human disease spinocerebellar ataxia type 1 (SCA1) offers another example where the presymptomatic mice showed reduced PC firing rate both in slices and in alert preparation, even before morphological alteration could be identified^81^. Interestingly, the gross motor behavior was not impaired at this stage of the disease, indicating that an important decrease in the firing rate (71 Hz in WT to 28 Hz in the SCA1 mice), which would logically reduce the PC GABAergic action on the DCN neurons, was not the prime inductor of ataxia. In addition, in spite of this PC frequency decrease, CS frequency was not significantly reduced in the SCA1 mice. This is consistent with the idea that the rate code alteration initiated by the PC output along the olivo-cerebellar loop is not able to explain ataxia.

In conclusion, the cell-specific genetic deletion of the BK channels in the PC, which is the sole output from the cerebellar cortex, appears to be the initial and determining element inducing an abnormal beta rhythm in both PC and DCN, LTD impairment and related ataxia. This study underlies the crucial need to understand oscillation as a way of neuronal communication and is certainly all the more important as a novel mutation in the *KCNMA1* gene has been associated with a cerebellar ataxic phenotype in a human patient^20^, presumably caused by a BK channel loss of function mutation.

## Materials and Methods

### Mice

PC-BK^−/−^ and WT littermate control mice with the same SV129xC57Bl6 background, used as experimental animals, were generated as follows^17^: Constitutive heterozygous BK L1/+ mice (SV129 background) were intercrossed with transgenic mice (C57BL/6 background) expressing the Cre recombinase under the control of the Purkinje protein 2 (PNP2) gene. The generation of this PNP2-Cre mouse line was described in detail by Barski *et al*.^82^. Progenies both carrying one BK L1 allele and being transgenic for PNP2-Cre were then crossed with mice carrying two loxP-flanked L2 alleles (BK L2/L2; SV129 background) of the BK gene KCNMA1 to obtain PNP2-Cre transgenic BK L2/L1 (PN-BK^−/−^) and PNP2-Cre transgenic BK L2/+ (PN-BK-Control) mice. The correct genotype was analyzed by PCR amplification as described previously^9,17^. Mice were bred and maintained at the animal facility of the Institute of Pharmacy, Department of Pharmacology, University of Tübingen, Germany. Either litter- or age-matched mice (at an age of 3 to 4 months) were randomly assigned to the experimental procedures with respect to the German legislation on animal protection. All animal procedures were approved by the University of Mons Ethics Committee and conducted in conformity with the European Union directive 609/86/EU. Every effort was made to minimize the number of animals and their discomfort. All animal procedures were approved by the University of Mons Ethics Committee and conducted in conformity with the European Union directive 609/86/EU. Every effort was made to minimize the number of animals and their discomfort.

### Surgical Preparation

Animals were prepared for chronic recordings of local field potential (LFP) and PC single-unit activity^33^ (Fig. 1). Mice were anesthetized with xylido-dihydrothiazin (Rompun©, Bayer, 10 mg/kg) and ketamine (Ketalar©, Pfizer, 100 mg/kg). Animals were administered an additional dose of xylido-dihydrothiazin (3 mg/kg) and ketamine (30 mg/kg) when they demonstrated agitation or marked increases in respiration or heart rate during the procedure. In addition, local anesthesia (0.5 mL of 20 mg/mL lidocaine and adrenaline [1:80000, Xylocaine©, Astra Zeneca]) was administered subcutaneously during the soft tissue removal. During surgery, two small bolts were cemented perpendicular to the skull to immobilize the head during the recording sessions, and a silver reference electrode was placed on the surface of the parietal cortex. To allow access to the vermis and the Crus I and II areas in the cerebellum, an acrylic recording chamber was constructed around a posterior craniotomy (2 × 2 mm) and covered with a thin layer of bone wax (Ethicon©, Johnson & Johnson).

In addition, bipolar silver stimulating electrodes were vertically implanted in the magnocellular division of the red nucleus (RN) (stereotaxic coordinate: 3.6 mm posterior, 0.5 mm lateral and 3.5 mm deep) and in the IO: 7.2 mm posterior, 0.25 mm lateral and 4.5 mm deep with respect to Bregma) following the method developed in the cat by Gruart and Delgado-Garcia^24^. Electrodes were aimed to the center of the mentioned structures and inserted contralaterally from the site of DCN recording following stereotaxic coordinates from the atlas of Franklin and Paximos^83^.

### Histological identification of the recording and stimulating sites

At the end of the experiment, electrolytic marks were placed in selected recording sites with quartz–platinum/tungsten microelectrodes (1 mA for 10 s). Then animals were deeply anesthetized with sodium pentobarbital (50 mg/kg, i.p.) transcardially perfused with saline and phosphate-buffered formalin. The location of the stimulating electrodes was made on serial 50-µm-thick sections of the brainstem and cerebellum mounted on glass slides and stained with Neutral Red.

### Electrical stimulation of the whisker region

Facial dermatomes of the whisker regions were electrically stimulated with a pair of small cutaneous needles inserted under the skin (inter-electrode distance 3–4 mm). Electrical stimulation consisted of a single square pulse, 0.2 ms in duration and <2 mA current intensity, delivered by an isolation unit (Isoflex, AMPI, Israel) connected to an analog pulse generator (Master 8, AMPI, Israel). The amplitude of the current delivered at intervals of 10 ± 3 s in the whisker region was adjusted in order to avoid overt movements and animal discomfort. The movement of the whisker was recorded by means of a home-made system using the A3515/16 BiCMOS linear Hall-effect sensors measuring the displacement of a micro-magnet of 0.003 g (1.0 mm diameter and 0.5 mm height), glued onto one whisker.

### Paradigm for inducing LTD

Paradigm used for inducing plasticity was the same as those developed by Marquez-Ruiz and Cheron^36^. It consisted of 15-min control situation in which single electrical stimuli were given at intervals of 10 ± 3 s in the whisker region. This period was immediately followed by 10 min of 8-Hz stimulation and then by 30 min of control during which the same single stimuli were applied at the same frequency as in the control situation. Electrophysiological responses to electrical stimulation in the whisker region were assessed by both the configuration of the LFP (which must show P1-N1-N2-P2-N3 components and by the identification of appropriate PC firing (modulated by spontaneous whisker movements and electrical stimulation). The P1/N1 component was taken to reflect the evoked activity in the mossy fibers of the granule cell (GC) layer while N2 and N3 are related to the activity of the synaptic link between the ascending axon of the granular and PC dendrite and between the parallel fiber and the PC dendrite, respectively. Data were analyzed off-line for amplitude and latency quantifications of N1, N2, and N3. For this analysis, 30 successive evoked field potentials were averaged to obtain one average data point every 5 min. The amplitude was computed by peak-to-peak measurements. For this calculation, negative peaks were compared to the trough of the preceding positive wave. When the P1 positive peak was not evident, we used the inflection point observed in the averaged trace for the N1 measurement. For N3 measurements, the amplitude was set as the difference between the positive peak between N2 and N3 and the negative peak of N3. Latency was determined as the time difference between stimulus onset and N1, N2, or N3 averaged peaks. For comparisons between animals, amplitude values for each one of the components of the LFP were normalized for statistical analysis.

### Single-unit and multiple-unit recordings in alert mice

Twenty-four hours after anesthesia, alert mice were restrained for the recording session. The dura was removed over the cerebellum to expose the tissue in the recording chamber. Recordings were performed in the Crus II area, and the depths of the electrodes were noted. To avoid unnecessary stress for the animals and movement artifacts, recording sessions were performed in a quiet room, when the animals were awake and calm. The alertness level was controlled by looking for the maintenance of whisking activity during the recording session. We used quartz–platinum/tungsten microelectrodes (1.2–3 MΩ) in a seven-channel Eckhorn microdrive (Thomas Recordings©, Giessen, Germany). All measures of impedance were made with a 1 kHz sinusoidal current and checked throughout the recording session. In the present study, the exploration were made with one or two microelectrodes separated from 250 µm (outer and shaft diameters of 80 µm and 25 µm, respectively). The microelectrode was mounted into a stretched elastic rubber tube to enable proper positioning via DC-micromotors (resolution of 0.27 μm).

Neural activity signal recordings were filtered at 100 Hz high-pass and 10 kHz low-pass. LFP and unitary electrical activities were stored digitally on a computer after conversion with an analog-digital converter (Power 1401, CED©, Cambridge, UK). The recorded data were digitized continuously at 20 kHz. Off-line analysis and illustrations were performed with Spike 2 CED software (CED©, Cambridge, UK).

The rhythmic frequency was defined as the reciprocal of the latency of the first peak in the autocorrelogram of SS firing (width = 1 s, bin size = 1.0 ms). Consequently, rhythmic frequency could not be determined on flat autocorrelograms. The strength of the rhythmicity was quantified with a rhythm index (RI) introduced by Sugihara *et al*.^84^. Briefly, peaks and valleys were recognized if their heights and depths exceeded the mean baseline level ± SD (measured at time lags of 250–300 msec). The RI was defined by the following formula: RI = *a1/z* + *b1/z* + *a2/z* + *b2/z* + …, in which *ai* (*i* = 1, 2, …) is the absolute value of the difference between the height of the *i*th peak and baseline level, b*i* (i = 1, 2, …) is the absolute value of the difference between the height of the *i*th valley and baseline level, and z was the difference between the height of the zero time bin and the baseline level. The strength of synchronicity between pair of PC was quantified by a synchronicity index (SI), measured on the cross-correlogram (width = 1 s, bin size = 1 ms). This was calculated by dividing the difference between the central peak of the cross-correlogram and the baseline level by the total number of spikes during the same period of time (the higher the synchronicity, the higher the synchronicity index). The central peak was defined as the highest peak in a 20 ms interval centered on the 0 ms time-bin^35^.

The regularity of the neuron was measured by the coefficient of variation (CV), defined as the quotient between the standard deviation and the mean of the interspike intervals. The recording and analysis of the different neurons were made the by an investigator blind to the genotype. Results are expressed and illustrated as mean ± S.D. and are considered significant if p < 0.05. All statistical analyses were performed using Statistica 7.0.

### Purkinje cells recordings

A neural signal was considered to originate from a PC when it presented two types of spiking activities: SS, characterized by a single depolarization (300–800 μs) that occurred between 20 and 200 Hz; and CS, characterized by an initial fast depolarization (300–600 μs), followed by smaller, and relatively constant wavelets. SS and CS were considered to originate from the same PC when a transient pause (>5.0 ms) in SS firing followed each CS. Recordings were analyzed when a stable signal was present for longer than 60 s. The configuration of the CS was studied by the measure of its duration, the number of spikelets, the intra CS frequency and the transient pause induced on the SS firing.

### Identification and recording of the DCN neurons

The deep cerebellar nuclei were located with the help of stereotaxic coordinates and the antidromic field potentials induced in interpositus and dentate nuclei by electrical stimulation of the red nucleus. Then, the region of interest was explored during this bipolar stimulation. The cathodic square pulses used for stimulation had a duration of 0.05 ms and an intensity ranging from 30 to 60 µA (below the threshold for eliciting overt movement). To be qualified as antidromically activated, a neuron had to meet three conditions: 1) it had to follow stimulation up to at least 200 Hz; 2) during a near-threshold stimulation the latency had to remain constant (±50 µs); 3) it had to show a collision of the antidromic spike with naturally occurring action potentials, demonstrable for spike-stimulus intervals as long as the antidromic latency period^43^. The differentiation between a somatic versus an axonic recording were made following the criteria adopted by Gruart and Delgado-Garcia^25^ which are based on the slope of the extracellular recording, the possibility of recording it over 100 µ; and the occurrence of an initial segment/somatodendritic break. In order to take into account the diversity of the neuronal type present in the DCN our analysis was not restricted to the neurons antidromically identified from the red nuclei. The neurons recorded in the close vicinity of an antidromically identified neurons were also studied and considered as a DCN neuron. The effects of the electrical stimulation of the IO on these DCN neurons were systematically analyzed by means of peri-stimulus time histogram (bin size of 2 ms) representing the neural discharge from 100 ms before to 400 ms after the repeated stimulation (n = 30). A firing rate response to a given stimulus was taken into account when their amplitude in the per-stimulus histogram represented more than the double or less than half of the mean resting value.

### Statistical analysis

Data were analyzed using a one–way ANOVA test and Bonferroni’s post-hoc test after assessing their normality by Kolmogorov-Smirnov test. For the analysis of the N1, N2 and N3 components during the 8 Hz LTD paradigm we used ANOVA for repeated measurements and Dunnett’s methods (post-hoc) when data were normally distributed and Friedman Repeated measurements analysis and Student-Newman-Keuls method (SNK) (post-hoc) when data were not normally distributed. Differences were considered significant at P < 0.05. Results are expressed as mean ± 1 SD.

## Electronic supplementary material

Supplementary Figure S1
