# Supplementary material for: Purkinje cell BKchannel ablation induces abnormal rhythm in deep cerebellar nuclei and prevents LTD
Source: Sci Rep. 2018 Mar 9;8:4220. doi: 10.1038/s41598-018-22654-6 (PMC5845018; doi:10.1038/s41598-018-22654-6)
Supplement: Supplementary file 1 — Supplementary Figure S1 [file 41598_2018_22654_MOESM1_ESM.pdf]

Supplementary information

Purkinje cell BK-channel ablation induces abnormal rhythm in deep cerebellar nuclei and prevents LTD

Guy Cheron [1,2*], Javier Márquez-Ruiz[3], Julian Cheron[2], Cynthia Prigogine[2], Claudia Ammann[3], Robert Lukowski [4], Peter Ruth[4], and Bernard Dan[2,5]

1. Laboratory of Electrophysiology, Université de Mons, Mons, Belgium

2. Laboratory of Neurophysiology and Movement Biomechanics, ULB Neuroscience Institut, Université Libre de Bruxelles, Brussels, Belgium

3. División de Neurociencias, Universidad Pablo de Olavide, Sevilla, Spain

4. Department of Pharmacology and Toxicology, Institute of Toxicology & Clinical Pharmacy, Universität Tübingen, Tübingen, Germany

5. Inkendaal Rehabilitation Hospital, Vlezenbeek, Belgium

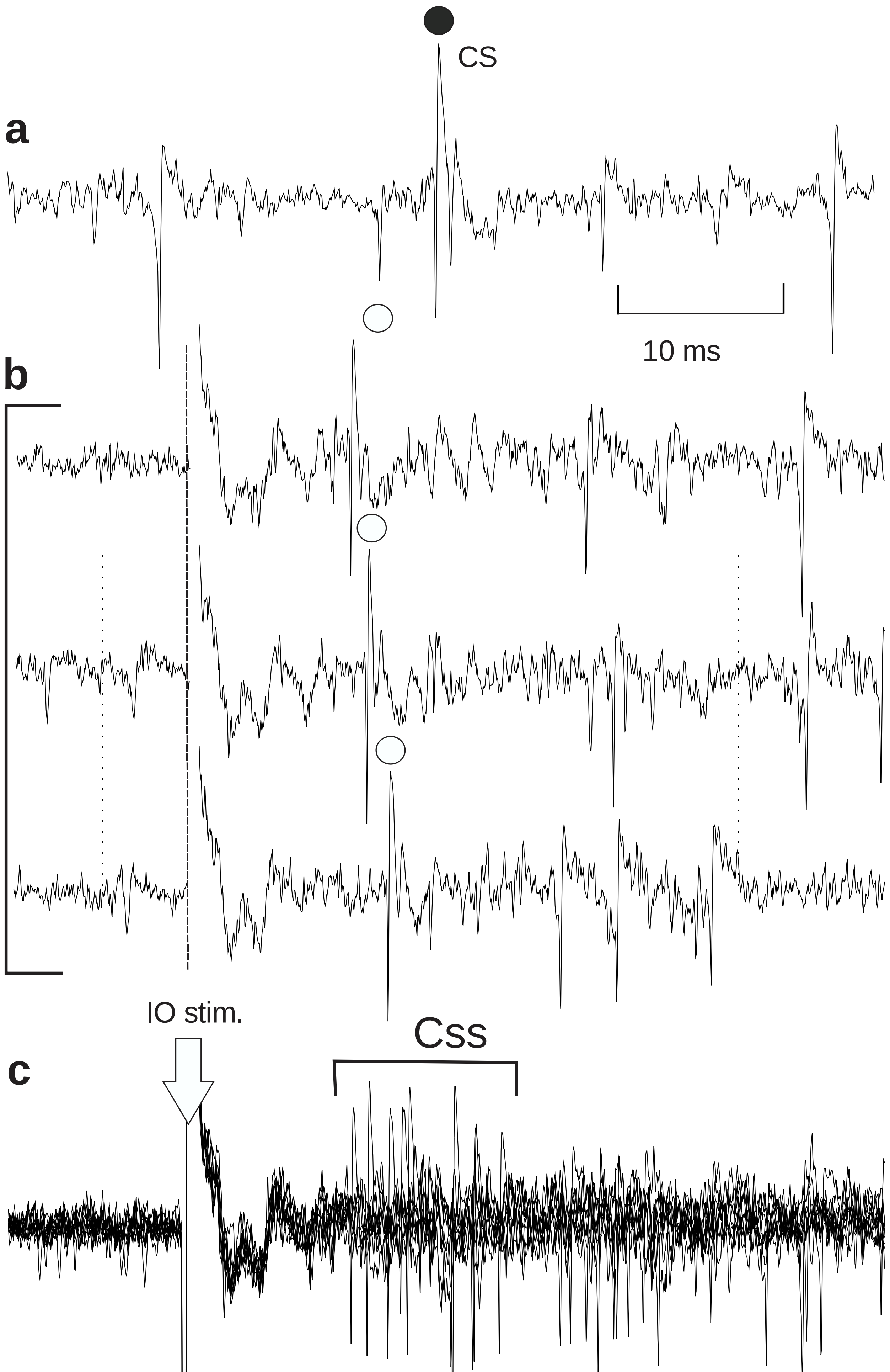
a
CS
10 ms
b
IO stim.
Css
c

Supplementary Figure S1. CS evoked by IO stimulation.(**a)** Configuration of spontaneous CS (marked by a black point) and recorded in a WT mouse.(**b)** Three examples of CS evoked by IO stimulation (marked by open circle). Note the same configuration of the spontaneous CS illustrated in **a** and the IO evoked CS illustrated in **b**. (**c)** Superimposition (n = 9) of PC firing evoked by IO stimulation (vertical arrow).
